# Longitudinal Analysis of the Intestinal Microbiota in Persistently Stunted Young Children in South India

**DOI:** 10.1371/journal.pone.0155405

**Published:** 2016-05-26

**Authors:** Duy M. Dinh, Balamurugan Ramadass, Deepthi Kattula, Rajiv Sarkar, Philip Braunstein, Albert Tai, Christine A. Wanke, Soha Hassoun, Anne V. Kane, Elena N. Naumova, Gagandeep Kang, Honorine D. Ward

**Affiliations:** 1 Division of Geographic Medicine and Infectious Diseases, Tufts Medical Center, Boston, MA, United States of America; 2 Departments of Public Health and Community Medicine, Tufts University School of Medicine, Boston, MA, United States of America; 3 Department of Integrative Physiology and Pathobiology, Tufts University School of Medicine, Boston, MA, United States of America; 4 Department of Gastrointestinal Sciences, Christian Medical College, Vellore, India; 5 Department of Computer Sciences, Tufts University School of Engineering, Medford, MA, United States of America; 6 Gerald J. and Dorothy R. Friedman School of Nutrition Science and Policy, Tufts University, Boston, MA, United States of America; Hospital for Sick Children, CANADA

## Abstract

Stunting or reduced linear growth is very prevalent in low-income countries. Recent studies have demonstrated a causal relationship between alterations in the gut microbiome and moderate or severe acute malnutrition in children in these countries. However, there have been no primary longitudinal studies comparing the intestinal microbiota of persistently stunted children to that of non-stunted children in the same community. In this pilot study, we characterized gut microbial community composition and diversity of the fecal microbiota of 10 children with low birth weight and persistent stunting (cases) and 10 children with normal birth weight and no stunting (controls) from a birth cohort every 3 months up to 2 years of age in a slum community in south India. There was an increase in diversity indices (P <0.0001) with increasing age in all children. However, there were no differences in diversity indices or in the rates of their increase with increasing age between cases and controls. The percent relative abundance of the Bacteroidetes phylum was higher in stunted compared to control children at 12 months of age (P = 0.043). There was an increase in the relative abundance of this phylum with increasing age in all children (P = 0.0380) with no difference in the rate of increase between cases and controls. There was a decrease in the relative abundance of Proteobacteria (P = 0.0004) and Actinobacteria (P = 0.0489) with increasing age in cases. The microbiota of control children was enriched in probiotic species *Bifidobacterium longum* and *Lactobacillus mucosae*, whereas that of stunted children was enriched in inflammogenic taxa including those in the Desulfovibrio genus and Campylobacterales order. Larger, longitudinal studies on the compositional and functional maturation of the microbiome in children are needed.

## Introduction

Malnutrition is a major cause of morbidity and mortality in children in low and middle-income countries [1]. Undernutrition is estimated to cause 3.1 million deaths annually or almost half of all child deaths (45%) in 2011 [1]. Children who survive often suffer from long-term consequences including growth and cognitive impairment [2–4]. Childhood malnutrition is part of a cycle of recurrent infections, impaired immunity, and worsening malnutrition, compounded by food insecurity and, likely, host genetic factors [5, 6]. Recently, alterations in the intestinal microbiome have been recognized as part of this cycle (reviewed in [7]). Elegant studies of children in Malawi [8, 9] and Bangladesh [10] have shown that moderate or severe acute malnutrition is causally linked to the gut microbiome and is associated with persistent immaturity of the gut microbiota. Secondary analysis of the data from two of these studies identified changes in the gut microbiota in severely stunted compared to stunted children [11]. A previous study from India reported changes in the gut microbiota of children of varying nutritional status including stunting [12]. However, there have been no primary longitudinal studies comparing the intestinal microbiota of persistently stunted children to that of non-stunted children in the same community.

Stunting or low height-for-age, the most common form of malnutrition, is very prevalent in resource-limited areas of the world and is considered the main indicator of childhood undernutrition [1]. Globally, over one quarter of children (165 million) under the age of 5 were stunted in 2011 and stunting contributes to over a million deaths in children in this age group [1, 13]. Most stunting occurs in the first 1000 days of life, which includes the time from conception to the end of the first two years of life [1]. The etiology of stunting is poorly understood. Several factors such as intrauterine growth retardation, neuroendocrine and hormonal factors, frequent diarrheal and other infections in early childhood, environmental enteric dysfunction, environmental toxins and host genetic factors are all implicated (reviewed in [6, 14–16]). Stunting is associated with low birth weight (LBW, < 2500 grams, [17]) [18–21] and is also associated with recurrent infections with infectious diarrhea the most important determinant [1, 22].

The largest number of stunted children in the world live in southern Asia [13]. In India, 48% of children under the age of 5 were estimated to be stunted in 2005–6 [23]. India had the highest numbers of LBW deliveries (7.5 million) globally in 2010 [24]. A longitudinal birth cohort study of children conducted between 2002 and 2006 in an urban slum community in Vellore, south India, found that 61% of children were stunted by the age of 3 years [19], with LBW significantly associated with stunting at 3 years of age (OR 3.63, 95% CI 1.36–9.70).

Recently, we completed a similar longitudinal birth cohort study in the same urban slum community in Vellore, India. In order to determine if there is an association between stunting and changes in the intestinal microbiota over time, we conducted a pilot longitudinal study of gut microbial communities in 10 children in the cohort with persistent stunting (cases) and compared them to that of 10 children with no stunting (controls), from 3 to 24 months of age. The aims of this study were to determine the effect of increasing age on the composition and diversity of the gut microbiota of all children and to determine if there were differences in the microbiota of cases compared to controls. Our hypothesis was that increasing age from birth to 24 months would have a significant impact on the composition and diversity of the gut microbiota in all children and that overall there would be differences in the gut microbial community composition between the cases and controls.

## Materials and Methods

### Study design and subjects

Data and stool samples from children enrolled in a birth cohort study designed to investigate immune responses to cryptosporidiosis from birth to 3 years of age in an urban slum community of Vellore, India were used for this study. The details of the study area and population, cohort recruitment and follow up of the parent study have been previously described [25]. Briefly, the study was conducted in four contiguous semi-urban slums with an area of ~ 2.2 sq Km and a population of 43,000 (2010 census) in the town of Vellore in the state of Tamil Nadu in India. Most people in the area are of a low socioeconomic status and live in homes consisting of brick walls with tile or cement roofs. The roads are paved but are lined with open drains. Water is supplied by the municipality to communal taps at intervals of 2–28 days depending on the season. Water is stored in wide-mouthed plastic or steel vessels inside the home and is not generally treated or boiled before use. The diet consists mainly of rice, lentils and vegetables. Although most families are not vegetarian, meat is not eaten daily, and the major protein sources are milk, eggs and lentils.

Four hundred and ninety seven children were enrolled in the parent study and were visited twice a week by trained field workers to document diarrhea and other morbidities. Of the 497 children 420 (84.5%) completed 2 years of follow up. Weight and length/height were measured monthly as described [25] and nutritional status defined by height-for-age (HAZ), weight-for-height (WHZ) and weight-for-age (WAZ) scores, using the 2006 WHO child growth standards as a reference [26]. Children were classified as stunted, wasted or underweight if their HAZ, WHZ or WAZ scores were greater than 2 standard deviations below the median of the WHO reference standard. Inclusion criteria for the parent study were 1) children born to families planning to stay in the study area for 3 years; 2) families willing to provide informed consent, participate in the study and have study personnel visit at home. Exclusion criteria were 1) children born to temporary residents of the area (those not planning to stay in the area for at least 3 years); 2) children with gross congenital anomalies including cardiovascular, renal or hepatic disease; 2) children with birth weight less than 1500 grams; 3) children with syndromic or serological evidence of HIV infection. Written informed consent was obtained from the parents or guardians of the children and approval obtained from the Christian Medical College and Tufts Health Sciences Institutional Review Boards for the parent study. Consent included the use of stored samples and data for future studies.

For the pilot study, data and stool samples from 2 groups of children in the cohort, 10 cases and 10 controls, were analyzed every 3 months from 3 to 24 months of age for a total of 8 time points and 160 samples. Persistent stunting was defined as an HAZ score greater than 2 SD below the median on at least 6 of 8 3-monthly time points. Severe stunting was defined as an HAZ score greater than 3SD below the median. Inclusion criteria for the cases were 1) low birth weight (<2500 grams), 2) persistent stunting and 3) at least one episode of diarrhea from birth to 24 months of age. Inclusion criteria for the controls were 1) normal birth weight; 2) no stunting at any of the 3-monthly time points and 3) no episodes of diarrhea from birth to 24 months of age.

### Stool DNA extraction and 16S rRNA gene amplicon generation and sequencing

Stool samples were transported to the laboratory on ice and frozen in aliquots at -80°C within 24 hours of collection. Overall community composition and diversity as well as relative taxon abundance were not shown to be significantly influenced by storage temperature or storage duration [27]. There was no difference in storage temperature or duration between cases and controls. DNA was extracted as described [28] using two successive rounds of bead beating with 0.1 mm zirconium–silica beads (Biospec Products, Bartlesville, OK, USA) followed by extraction with a QIAamp DNA Stool Minikit (Qiagen, Germantown, MD). The V4 region of the 16S rRNA gene was PCR amplified based on previously described protocols [29]. Briefly, amplicons were generated using V4 forward primer 515F5’ GTGCCAGCMGCCGCGGTAA and reverse primer 806R3’ GGACTACHVGGGTWTCTAAT,’ each attached to the appropriate Illumina adapter sequences. The reverse primer contained a unique 12 base Golay barcode sequence. PCR amplifications were performed in triplicate using a HotStarTaq Master Mix kit (Qiagen) and the following PCR conditions: 95°C for 2 minutes, 20 cycles of 94°C for 30 seconds, 52°C for 45 seconds, 65°C for 5 minutes and extension at 65°C for 15 minutes. A negative control was run on each PCR reaction plate: reactions yielding no amplicons or those in which the negative controls were positive were repeated. PCR products were purified using a QIAquick PCR Purification Kit (Qiagen). The DNA concentration of amplicons was determined using a Quant-iT assay (Invitrogen, Carlsbad, CA). Amplicons were then pooled in equimolar concentrations and purified using Agencourt Ampure XP beads (Beckman-Coulter, Beverly, MA). Purified, pooled amplicons were subjected to paired-end (150 x 150bp) sequencing on an Illumina MiSeq according to standard Illumina protocols at the Genome Research Laboratory of the Virginia Bioinformatics Institute at Virginia Tech University. Paired-end reads of 150 bp in length in each direction were generated with an approximately 46 bp overlap when combined.

### Computational and statistical analyses

Computational analyses were performed using QIIME Version 1.8 (http://www.qiime.org) [30]. Statistical analyses were performed in R (http://www.r-project.org/). Quality filtering was performed using default settings in QIIME. Reads were assigned to operational taxonomy units (OTUs) based on 97% identity using the closed-reference OTU algorithm (pick_closed_reference_otus.py) with taxonomy assigned using the Greengenes predefined taxonomy map [31]. Reads that did not match a reference sequence were discarded. Alpha diversity, defined as diversity within a given community that is characterized using the total number of species (species richness), the relative abundances of the species (species evenness), or indices that combine these two dimensions [32] including Shannon Diversity, Equitability, Number of Observed OTUs, Chao and Phlyogenetic Diversity (PD) were computed in QIIME. Beta diversity, defined as partitioning of diversity among communities which is characterized using the number of species shared between communitieswas determined by principal coordinates analysis (PCoA) of weighted and unweighted UniFrac distances in QIIME and differences were determined using a 2-sample t-test with 1000 Monte Carlo permutations and Bonferroni corrections.

The Linear Discriminant Analysis Effect Size (LEfSe) algorithm was used to identify differentially abundant taxa using default parameters http://huttenhower.sph.harvard.edu/galaxy/) [33]. Since this was an exploratory study, corrections for multiple testing were not performed [34].

To estimate the age-specific change in outcomes and the degree of change at a group level, accounting for potential serial autocorrelation in the repeated measurements, the separate linear mixed effects models were fitted to individual trajectories. Each model described outcomes as a function of time with birth weight and gender as covariates and age in days as a random variable using the lmer-function of the lme4 R package. Based on the model results the 3-monthly rate of change for each outcome along with lower and upper limits of their 95% Confidence Intervals (CI) were estimated.

## Results

### Sociodemographic, clinical and nutritional characteristics

There were no significant differences in gender, gestational age, socioeconomic status, age of weaning or number of children who were exclusively breast fed for 6 months, between the case and control groups (Table 1). All children were breast-fed, though for varying periods of time. None of the children in either group were born pre-term and none were born by C-section. Based on the inclusion criteria, control children had significantly higher birth weights than cases and did not experience any diarrheal episodes in the first 24 months of life. All case children experienced at least one episode of diarrhea with a total of 66 episodes and an average of 6.6 episodes/child over the first 24 months of age (S1 Fig). One episode of diarrhea was due to *Cryptosporidium*. All other episodes of diarrhea were of unknown etiology.

**Table 1:** Baseline socio-demographic and clinical data in cases and controls.

Parameter	Controls	Cases	P
**Gender, male (%)**	3/10 (30%)	3/10 (30%)	1.00^3^
**Gestational age in wks, median (IQR)**^1^	40 (39.7–40.8)	37.8 (36.8–40.2)	0.19^4^
**Birth weight in Kg, median (IQR)**	2.9 (2.7–3.3)	2.1 (1.9–2.3)	**0.0002**^4^
**Socioeconomic status**^2^			
**Low**	6/10 (60%)	6/10 (60%)	1.00^3^
**Middle**	4/10 (40%)	4/10 (40%)	1.00^3^
**Age of weaning in months**	3.8 (1.8–5.6)	3.3 (1.4–5.2)	0.762^3^
**Exclusive breast feeding for 6 mths**	1/10 (10%)	2/10 (20%)	1.00^3^
**Exclusive breast feeding for 3 mths**	5/10 (50%)	4/10 (40%)	0.5^3^
**No. with diarrhea from 0 to 24 mths**	0/10 (0%)	10/10 (100%)	**<0.0001**^3^
**No. of diarrheal episodes from 0 to 24 mths**	0	66	-
**No. treated with antibiotics from 0 to 24 mths**	0/10 (0%)	9/10 (90%)	**<0.0001**^3^
**No. of episodes of antibiotic use from 0 to 24 mths**	19	81	**0.01**^4^
**No. with diarrhea from 0 to 3 mths**	0/10 (0%)	3/10 (30%)	0.2105^3^
**No. of diarrheal episodes from 0 to 3 mths**	0	8	-
**No. treated with antibiotics from 0 to 3 mths**	1/10 (10%)	4/10 (40%)	0.3034^4^
**No. of episodes of antibiotic use from 0 to 3 mths**	1	6	0.09^4^

^1^information missing for one subject

^2^assessed using a 5 point scale modified from the Kuppuswamy scale

^3^Fisher’s exact test

^4^Mann-Whitney test. Statistically significant P values (P <0.05) are in bold type

All 10 cases and 6 of the 10 controls were treated with antibiotics mainly for diarrhea or respiratory illness (S1 Fig). The most commonly used antibiotic was sulfamethoxazole/trimethoprim. Amoxicillin, cefixime, cephalexin, norfloxacin and metronidazole were also used. The case children were treated with antibiotics for a total of 33 times for diarrhea and 48 times for other conditions with an average of 8.1 episodes of antibiotic use per child during the first 24 months of age (Table 1 and S1 Fig). Since none of the controls had diarrhea, they were not treated with antibiotics for that condition; however, 6 of 10 control children received antibiotics for other conditions for a total of 19 times with an average of 1.9 episodes of antibiotic use per child during the first 24 months of life (Table 1 and S1 Fig).

**Fig 1:**
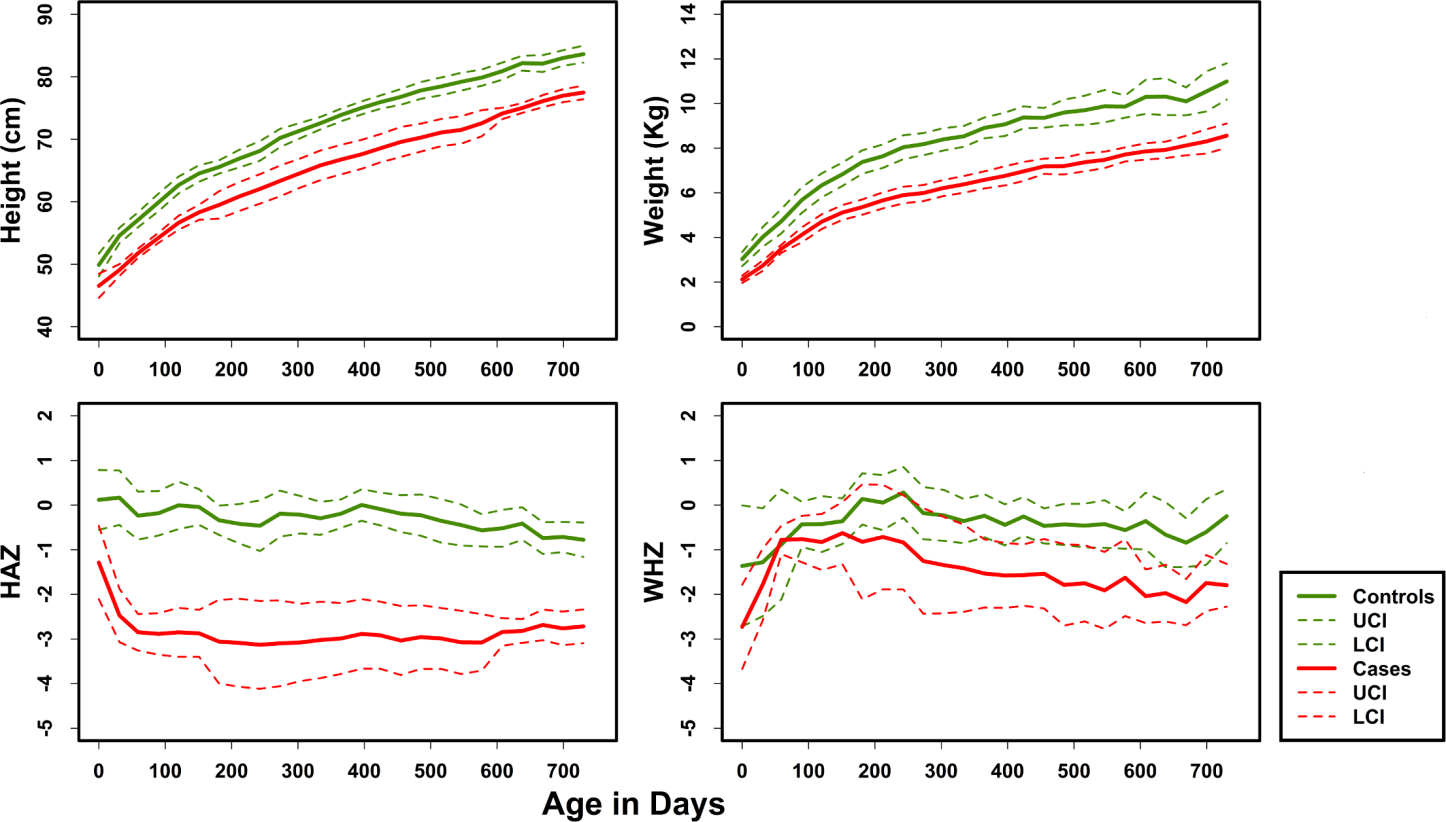
Growth trajectories in cases and controls: Mean height, weight, HAZ and WHZ scores and corresponding 95% confidence intervals (CI) were plotted over time from birth to 24 months of age. HAZ, Height for Age, WHZ, Weight for Height, UCI, Upper Confidence Interval, LCI, Lower Confidence Interval.

The mean height and weight gain with 95% CI for children in both groups over the first 24 months of age are shown in Fig 1A and 1B. Control children had significantly greater weights and heights at each 3-monthly time point than case children (S1A Table). However, it should be noted that the heights and weights of the control children were also lower than the WHO standards for the region (not shown). The mean HAZ and WHZ scores with 95% CI are shown in Fig 1C and 1D. Although the average HAZ, WAZ and WHZ scores of control children at any of the time points were all below 0, they were significantly greater than those of the cases at each of the 3-monthly time points (S1B Table). Based on the inclusion criteria, none of the controls were stunted at any time point whereas all case children were persistently stunted (S1 Fig, S1B Table). One case was stunted at birth; the other 9 were not. However, by 3 months, 9 case children were stunted (4 of them severely). By 24 months, 9 case children remained stunted (5 severely). The most growth faltering occurred in the first 3 months of age in cases. Based on the inclusion criteria, none of the controls had diarrhea whereas 3 of the cases had diarrhea during the first 3 months of age (Table 1). There were no significant differences among cases and controls in the number of children who were exclusively breast fed for 3 months or in the number of children who were treated with antibiotics or in the total number of episodes of antibiotic use during this time (Table 1).

### Intestinal microbiota

Sequencing of the V4 region of the 16S rRNA gene yielded a total of 5,928,140 sequences, with a sequencing depth of 20,000 to 75,000 (mean = 37,000 and median = 34,100) reads per sample. For comparative analysis, sequence numbers were normalized by rarefaction to 20,000, the sequence count of the sample with the smallest number of sequences. We analyzed these sequences to assess stool microbial community composition and diversity from control and case children from 3 to 24 months of age.

**Fig 2:**
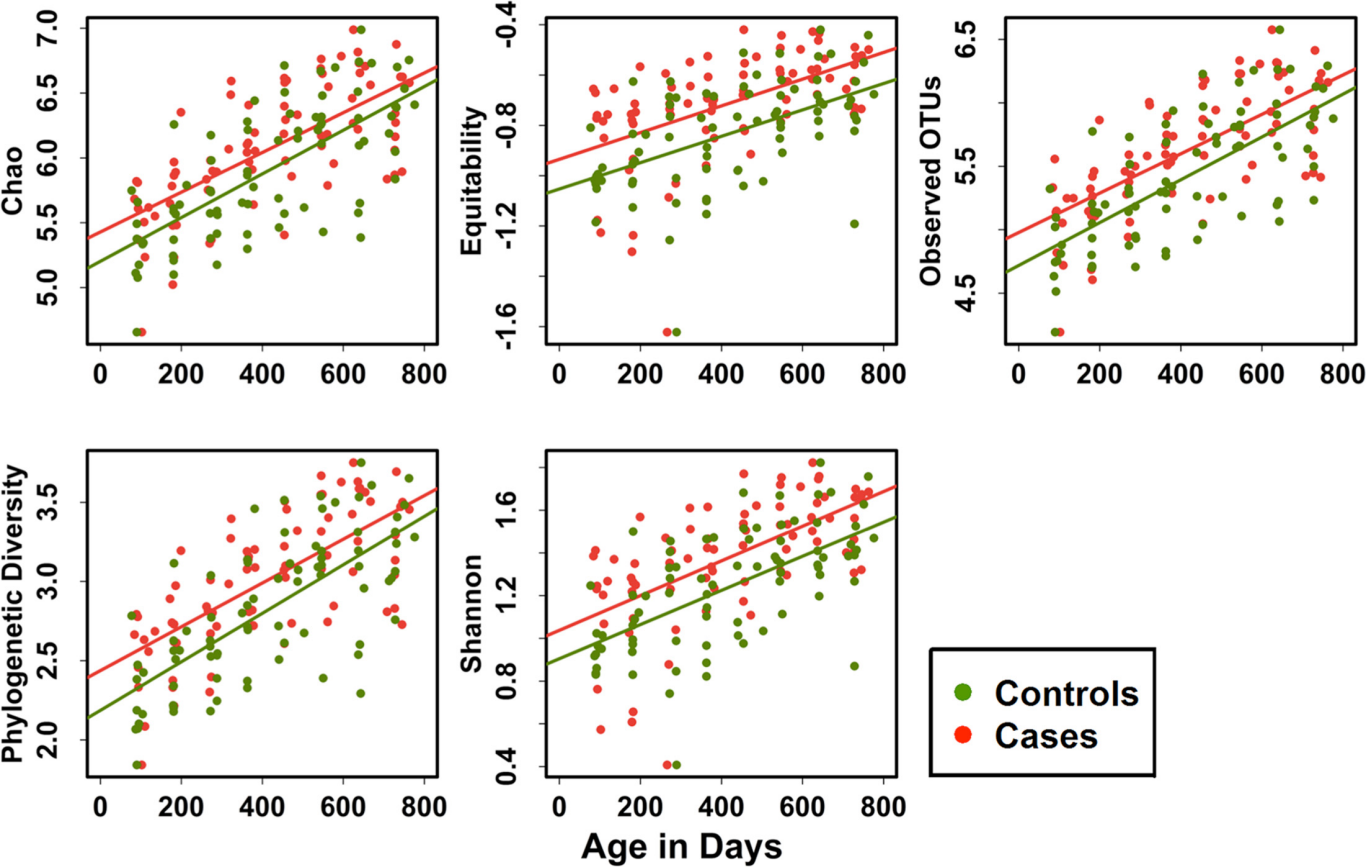
Alpha diversity indices in cases and controls: Alpha diversity indices (Chao, Equitability, observed OTUs, Shannon and Phylogenetic Diversity (PD) were computed in QIIME. regression model showing the increase in individual diversity indices over time from 3 to 24 months of age.

To determine the effect of increasing age on the alpha diversity of the gut microbiota of case and control children, we used a linear mixed effects model, adjusted for gender and birth weight. Fig 2 shows a steady increase in all 5 alpha diversity indices (Observed OTUs, Chao, Shannon, Equitability, Phylogenetic Diversity (PD) in all children with increasing age. Using model estimation of the change in diversity indices associated with 3 months of age we found that the increase in diversity was highly significant for all children as well as those in the case and control groups separately for all diversity indices examined (P <0.0001). Although the rates of increase (shown as relative risk in Table 2) for all diversity indexes were greater in cases compared to controls, the incremental increases for individual indices were not significant between the groups.

**Table 2:** Age related effect on alpha diversity indices*.

All	3 RR Months	UCI	LCI	P
**Chao1**	0.1258	0.1624	0.0891	**<0.0001**
**Observed OTUs**	0.1298	0.1681	0.0915	**<0.0001**
**Shannon**	0.0609	0.0840	0.0377	**<0.0001**
**Equitability**	0.0376	0.0552	0.0200	**<0.0001**
**Phylogenetic Diversity**	0.1141	0.1455	0.0827	**<0.0001**
**Controls**				
**Chao1**	0.1255	0.1597	0.0912	**<0.0001**
**Observed OTUs**	0.1295	0.1646	0.0945	**<0.0001**
**Shannon**	0.0606	0.0785	0.0428	**<0.0001**
**Equitability**	0.0375	0.0506	0.0243	**<0.0001**
**Phylogenetic Diversity**	0.1139	0.1428	0.0850	**<0.0001**
**Cases**				
**Chao1**	0.1381	0.1769	0.0994	**<0.0001**
**Observed OTUs**	0.1400	0.1811	0.0988	**<0.0001**
**Shannon**	0.0729	0.1007	0.0451	**<0.0001**
**Equitability**	0.0476	0.0693	0.0258	**<0.0001**
**Phylogenetic Diversity**	0.1241	0.1555	0.0927	**<0.0001**

*Analyzed using a linear mixed effects regression model, constructed for each index and adjusted for birth weight and gender. RR, relative risk; UCI, upper confidence interval; LCI, lower confidence interval. Statistically significant P values (P <0.05) are in bold type. The regression coefficients associated with age are expressed as relative risks (RR), or the degree of change in each index that occurred over a 3-month period. To illustrate the degree of uncertainty, the estimates of RR are accompanied by values of the lower and upper confidence intervals (LCI and UCI, respectively). For each model we also provide information related to statistical significance (p-value) for the estimates with respect to a hypothesis that detected associations likely are not by chance. For example, interpretation of the first row of results can be read as follows: The Chao1 diversity index is likely to increase significantly by 0.1258 within the 3 month time interval.

S2 Fig shows the effect of increasing age on the PD index (which measures the sum of the branch length of all the branches on a phylogenetic tree) of the microbiota of individual children in both groups in relation to diarrheal episodes, antibiotic use and age of weaning. There was considerable variation in the rate of increase in PD between both groups and no clear pattern could be identified between the groups. There were no significant differences in alpha diversity (within sample diversity) indices (Observed OTUs, Chao, Shannon, Equitability and Phylogenetic Diversity) between case and control children at each of the 3-monthly time points up to 24 months of age (S2 Table).

We analyzed beta diversity (between sample diversity) of microbial communities in all children by unsupervised clustering using principal coordinates analysis (PCoA) of UniFrac distance matrices [35] at each 3-monthly time point. There was a significant increase in UniFrac distance in cases compared to controls (P = 0.01) at the 12-month time point, but not at other time points (S3 Fig).

**Fig 3:**
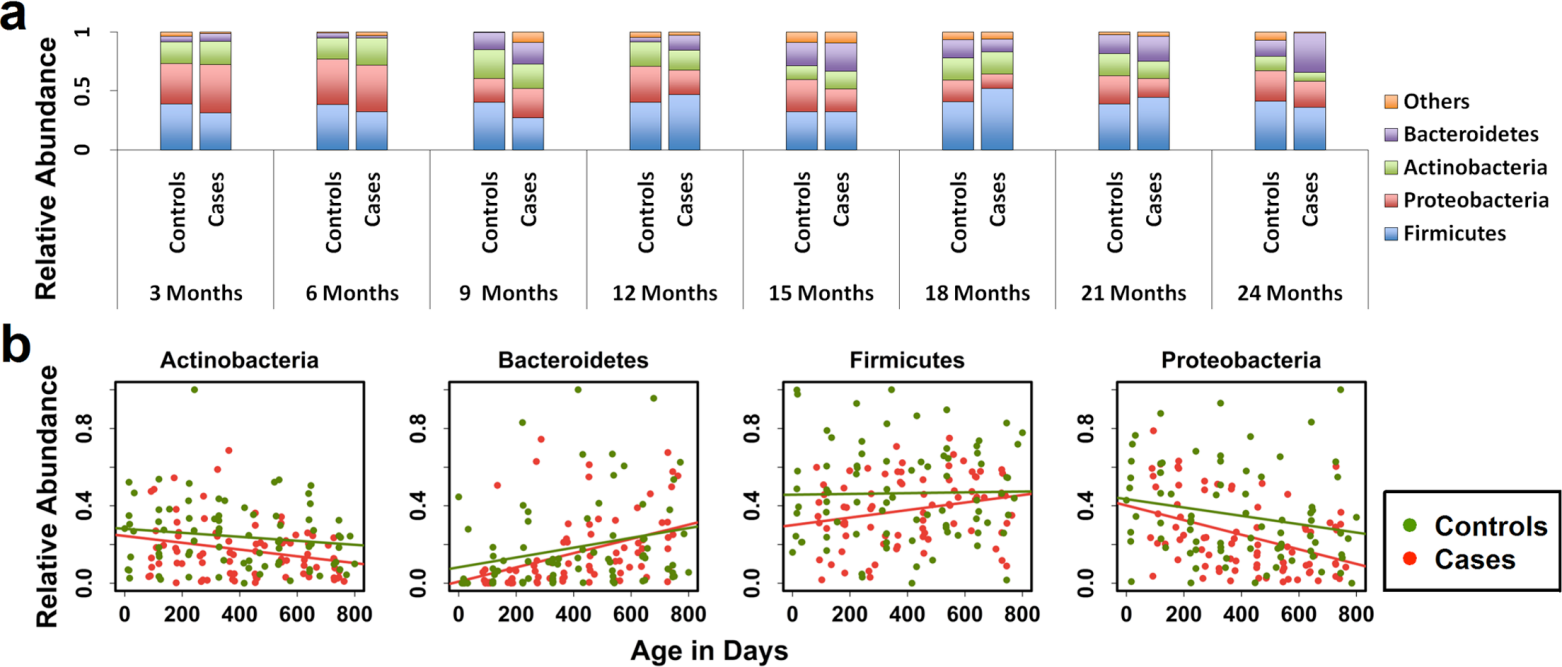
Relative abundance of major phyla in cases and controls. **a:** Average relative abundance of major phyla at each 3 monthly time point. **b:** inear regression model showing the change in relative abundance of each major phylum over time from 3 to 24 months of age.

The most abundant taxa at the phylum level in both groups overall were Firmicutes (38.6%), Proteobacteria (25.89%), Actinobacteria (17.5%), Bacteroidetes (13.8%) and Verrucomicrobia (2.6%) (Fig 3A). The relative abundance of Bacteroidetes was significantly higher in cases at 12 (P = 0.043) and approaching significance at 24 (P = 0.0524) months of age compared to controls (S2 Table). However, there were no significant differences in the relative abundance of any of the other phyla between cases and controls at each of the 3-monthly time points. We used a linear mixed effects model, adjusted for gender and birth weight to determine the effect of increasing age on the relative abundance of the major phyla (Fig 3B). Using model estimation of the change in relative abundance, we found that the relative abundance of Bacteroidetes significantly increased over time for all children combined (P = 0.0380) as well as controls (P = 0.0258) and cases (P <0.0001) separately (Table 3). The decrease in relative abundance of Proteobacteria (P = 0.0004) and Actinobacteria (P = 0.0489) with increasing age was significant only in the cases, with a trend towards a significant decrease in that of Proteobacteria in all children (P = 0.0613), and in controls (P = 0.0768). There was no significant change in the relative abundance of Firmicutes with increasing age in either group, though there was a trend towards a significant increase in cases (P = 0.0546).

**Table 3:** Age-related effect on the relative abundance of major phyla*.

	3 RR Month	UCI	LCI	P
**All**				
**Actinobacteria**	-7.97E-03	6.23E-03	-2.23E-02	0.2694
**Bacteroidetes**	1.62E-02	3.17E-02	7.43E-04	**0.0380**
**Firmicutes**	1.51E-03	1.79E-02	-1.49E-02	0.8550
**Proteobacteria**	-1.71E-02	9.95E-04	-3.52E-02	*0*.*0613*
**Controls**				
**Actinobacteria**	-7.89E-03	4.26-E03	-2.00E-02	0.1987
**Bacteroidetes**	1.60E-02	3.03E-02	1.79E-03	**0.0258**
**Firmicutes**	1.63E-03	1.96E-02	-1.64E-02	0.8574
**Proteobacteria**	-1.69E-02	2.01E-03	-3.58E-02	*0*.*0768*
**Cases**				
**Actinobacteria**	-1.61E-02	8.45E-05	-3.23E-02	**0.0489**
**Bacteroidetes**	3.31E-02	4.94E-02	1.67E-02	**<0.0001**
**Firmicutes**	1.75E-02	3.56E-02	-5.26E-04	*0*.*0546*
**Proteobacteria**	-3.39E-02	-1.49E-02	-5.29E-02	**0.0004**

*Analyzed using a linear mixed effects model, adjusted for birth weight and gender. Please see footnote to Table 2 for explanation of results. RR, relative risk; UCI, upper confidence interval; LCI, lower confidence interval. Statistically significant P values (P <0.05) are in bold type and those approaching statistical significance are in italics.

Overall the most abundant genera were *Bifidobacterium* (14.85%), *Streptococcus* (10.92%), *Prevotella* (6.65%), *Bacteroides* (6.05%), *Lactobacillus* (3.03%), *Veillonella* (2.69%), *Akkermansia* (2.65%), *Megasphaera* (1.79%), *Eubacterium* (1.20%) and *Haemophilus* (1.13%) (S4 Fig).

**Fig 4:**
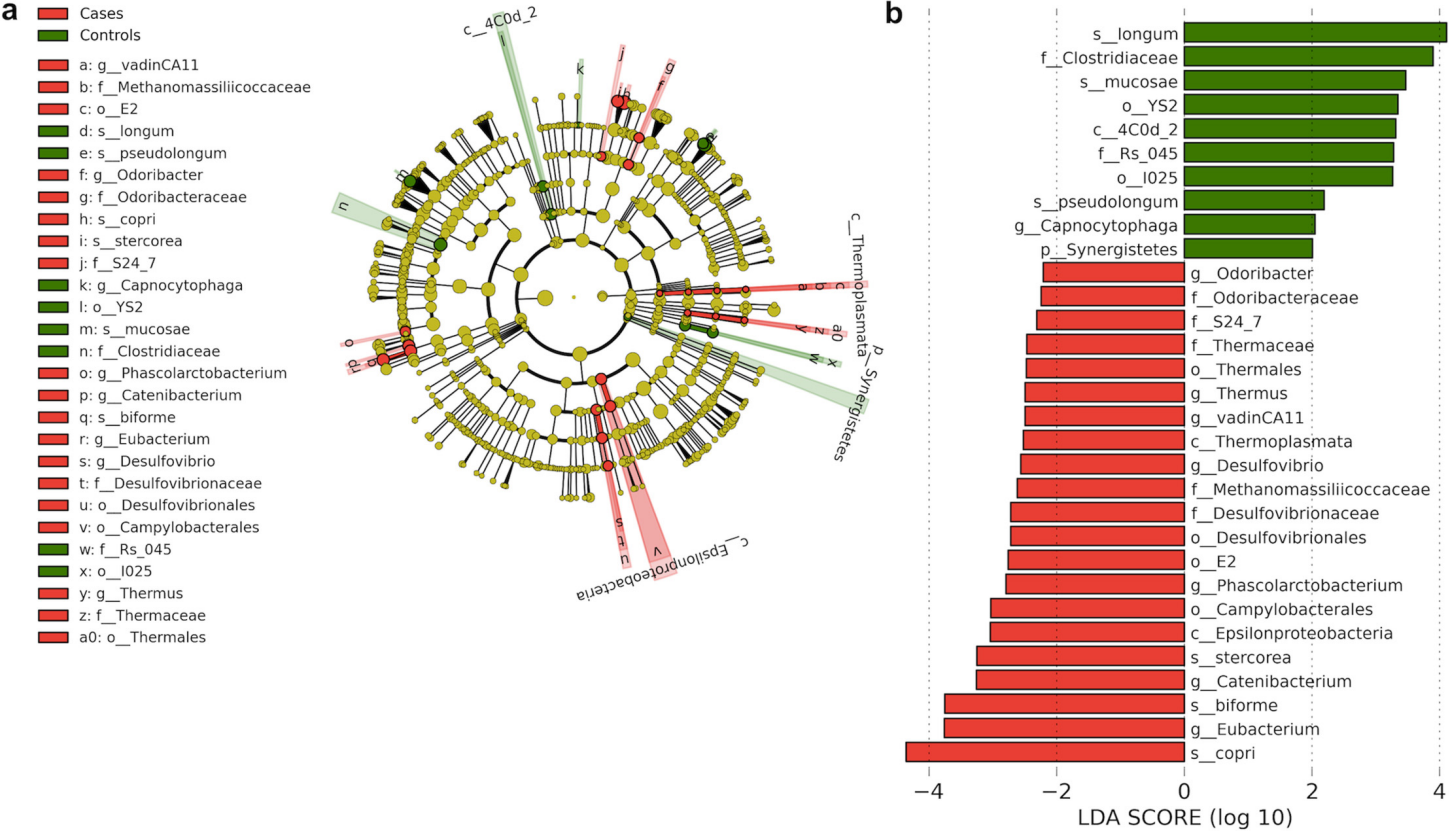
Differentially abundant taxa between cases and controls. **a:** Linear Discriminant Analysis Effect Size (LEfSe) cladogram of differentially abundant taxa in cases and controls [phylum (p), class (c), order (o), family (f), genera (g), species (s)]. **b:** Linear Discriminant Analysis (LDA) scores of differentially abundant taxa in cases and controls. The LDA score indicates the effect size and ranking of each differentially abundant taxon.

To determine whether any taxa at different taxonomic levels were enriched or depleted in the control and case groups, we used the LEfSe algorithm, which identifies genomic features characterizing the differences between two or more biological conditions [33]. Since children in both groups were the same age at each time point, we analyzed all data for each group using LEfSe. Taxa that were enriched in the controls (and thus depleted in the cases) included *Bifidobacterium longum*, *Bifidobacterium pseudolongum* and *Lactobacillus mucosae* species and the Clostridiaceae family (Fig 4A and 4B). Conversely, taxa enriched in the cases (and depleted in the controls) included *Prevotella stercorea*, *Prevotella copri* species, Desulfovibrio and Catenibacterium genera and Campylobacterales order. We used the linear mixed effects model to determine if there was an effect of increasing age on the relative abundance of taxa that were enriched or depleted in each group. There was a significant decrease in the relative abundance of *Bifidobacterium longum* (P = 0.0007) and *Bifidobacterium pseudolongum* (P = 0.0161) and a significant increase in that of Clostridiaceae (P = 0.0093), Eubacterium and *Eubacterium biforme* with increasing age in both controls (P = 0.0497 for both) and cases (P = 0.0047 for both) and in that of Clostridiaceae (P = 0.0150) in cases.

## Discussion

In this longitudinal study we investigated the composition and diversity of gut microbial communities in persistently stunted children in a birth cohort compared to non-stunted children in the same cohort, living in the same community. We found that there were age-related changes in the composition and diversity of the gut microbiota of all children, with differences in the relative abundance with increasing age in specific taxa between persistently stunted and non-stunted children. The gut microbiota of stunted children was enriched in inflammogenic taxa whereas that of non-stunted children was enriched in probiotic bacterial species.

Previous studies (including an earlier birth cohort study of children from the same site where the current study was conducted [19]) have found that low birth weight is a risk factor for stunting [18–21]. Stunting is also associated with recurrent diarrheal infections [1, 22]. In the present study, among the 420 children who completed 2 years of follow up, only 27 children (6.42%) did not have any episode of diarrhea and only 15 (3.57%) children did not have any episode of diarrhea and were not stunted on any of the 8 3-monthly time points. The control group was selected from among these children and therefore represents a very small subpopulation that is not representative of the community. However, we felt that a control group from the same community, with similar dietary practices and environmental and hygiene conditions was better than a control group of healthy children from outside the community.

Low birth weight and at least one episode of diarrhea were inclusion criteria for the case group in the current study. All children in this group had one or more episodes of diarrhea and all of them were treated with antibiotics for diarrhea or other conditions. Six children in the control group were treated with antibiotics for conditions other than diarrhea. Although most antibiotic use occurred after 6 months of age, most case children were stunted by this age. Recent studies in the same birth cohort of children as in the present study found that exposure to antibiotics early in life were associated with increased rates of diarrhea in early childhood [36], but that there were no significant associations between antibiotic use and growth faltering [37]. Diarrheal infections and antibiotic use, both of which are very common in India [38, 39] are known to significantly alter the composition and diversity of the gut microbiota [40–42]. However, it is not possible to separate out the possible effects of diarrhea and antibiotic use from those of stunting on the gut microbiota of children in this study.

Breast-feeding including the duration of exclusive breast-feeding, cessation of breast-feeding, age of weaning and diet are also known to impact the gut microbiota [43–46]. In our study, all children were breast-fed and there were no significant differences in the number of children who were exclusively breast fed for 3 months or for 6 months or the age of weaning between stunted and non-stunted children. We were therefore not able to identify an association between breast-feeding and the gut microbiota in stunted or control children.

Most of the growth faltering in the cases occurred in the first 3 months of life. There were no significant differences in rates of exclusive breast feeding, diarrheal episodes or antibiotic use between the cases and controls during this time. In addition, there were no differences in the composition or diversity of the fecal microbiota between the groups, suggesting that early stunting in this community may not be associated with changes in the microbiota and that other factors such as maternal nutrition or intrauterine growth may play a role.

Several studies have reported on the composition and diversity of the gut microbiota of infants and young children [8, 44, 47–50]. Although the methods (next generation sequencing, microarray, real time PCR), sequencing platforms (Roche 454, Illumina MiSeq or HiSeq), V regions targeted for amplicon generation (V4, V3-V5, V4-V5), health status (healthy, malnourished, with enteric infections), age range (0 to 6 years), geographic location (USA, Italy, Sweden, Malawi Burkina Faso, Bangladesh, India) and breast-feeding, weaning and dietary practices of the children studied have varied, making comparisons difficult, it is universally agreed that the microbiota of young children is different from that of adults, varying considerably with increasing age and reaching adult levels of maturity by 1 to 3 years.

As in previous studies which examined the impact of increasing age on the gut microbiota [10, 44, 48, 49], our study shows that there was a significant increase in alpha diversity with increasing age in all children in both groups. However, whereas alpha diversity plateaued, reaching adult levels by one year of age in children in Sweden [44], in our study, alpha diversity continued to rise till 2 years of age. Yatsunenko et al also showed that alpha diversity continued to increase till 3 years of age in Malawian, Amerindian and US children although the microbiota of US children was the least diverse [49].

In our study, we found that overall Firmicutes and Proteobacteria were the most abundant phyla, followed by Actinobacteria and Bacteroidetes. Surprisingly, this pattern of relative abundance is similar to that reported in children from the United States [47, 48] and Italy [46] where Proteobacteria and Firmicutes predominated. In contrast, Bacteroidetes and Actinobacteria were the predominant phyla in Burkina Faso [46], Firmicutes and Bacteroidetes in Sweden [44] and Actinobacteria and Firmicutes in Canada [51].

Using the LEfSe algorithm, we identified specific taxa that were enriched or depleted in the microbiota of children in each group. The taxa enriched in the control children included the probiotic species *Bifidobacteria longum*, which are associated with healthy gut microbiota of breast-fed infants [52, 53] as well as *Lactobacillus mucosae*, another potential probiotic species [54]. The decrease in the relative abundance of *B*. *longum* and *B*. *pseudolongum* with increasing age in all children is consistent with decreasing rates of breastfeeding, since these species use milk oligosaccharides as substrates [55]. The studies in Bangladesh [50] and Malawi [9] both found that *Bifidobacterium longum* and *Faecalibacterium prausnitzii* were among the most age-discriminatory taxa in healthy infants and children in these countries. Both these species were identified in cases and controls in our study. *B*. *longum* was enriched in controls compared to cases, but there was no difference in the relative abundance of *F*. *prausnitzii* between children in each group. The relative abundance of *B*. *longum* decreased (Table 4), whereas that of *F*. *prausnitzii* increased with increasing age in all children (RR = 1.63E-03 UCI = 2.90E-03, LCI = 3.55E-04, P = 0.0113, see footnote to Table 2 for explanation).

**Table 4:** Age-related effect on the relative abundance of differentially abundant taxa*.

All	3 RR Months	UCI	LCI	P
**s_*Bifidobacterium longum* (A)**	-1.96E-02	-8.14E-03	-3.10E-02	**0.0007**
**s_*Bifidobacterium pseudolongum* (A)**	-2.01E-04	-3.58E-05	-3.67E-04	**0.0161**
**o_I025, f_Rs_045 (TM7)**	1.49E-03	3.02E-03	-3.99E-05	*0*.*0538*
**g_Catenibacterium (F)**	2.30E-03	4.62E-03	-3.07E-05	*0*.*0507*
**f_Clostridiaceae (F)**	4.99E-03	8.78E-03	1.19E-03	**0.0093**
**Controls**				
**s_*Bifidobacterium longum* (A)**	-1.96E-02	-8.47E-03	-3.07E-02	**0.0005**
**s_*Bifidobacterium pseudolongum* (A)**	-2.01E-04	-2.62E-05	-3.76E-04	**0.0228**
**g_Eubacterium, s_*Eubacterium biforme* (F)**	2.32E-03	4.65E-03	-2.02E-05	**0.0497**
**Cases**				
**s_*Bifidobacterium longum* (A)**	-1.94E-02	-7.15E-03	-3.16E-02	**0.0017**
**s_*Bifidobacterium pseudolongum* (A)**	-2.02E-04	-4.47E-05	-3.60E-04	**0.0110**
**g_Eubacterium, s_*Eubacterium biforme* (F)**	5.56E-03	9.45E-03	1.66E-03	**0.0047**
**g_Catenibacterium (F)**	2.53E-03	4.55E-03	5.10E-04	**0.0131**
**f_Clostridiaceae (F)**	3.09E-03	5.61E-03	5.75E-04	**0.0150**

*identified by LEfSe at all time points combined. Analyzed using a linear mixed effect model adjusted for birth weight and gender. Please see footnote to Table 2 for explanation of results. Taxa whose relative abundance decreased over time are indicated with a negative sign. RR, relative risk, UCI, upper confidence interval, LCI, lower confidence interval. p, phylum; o, order, c, class, f, family, g, genus, s, species; Phyla to which specific taxa belong are indicated in brackets; A, Actinobateria, F, Firmicutes. Statistically significant P values (P <0.05) are in bold type and those approaching statistical significance are in italics.

Taxa enriched in stunted (case) children included inflammogenic genera such as Desulfovibrio which are enriched in patients with inflammatory bowel disease [56] as well as the Desulfovibrionaceae family which contains the sulfite-reducing pathobiont, *Bilophila wadsworthia* that is associated with the microbiota of children with Kwashiorkor [8] and with a pro-inflammatory TH 1 immune response and colitis in IL-10-deficient mice [57]. The microbiota of stunted children was also enriched in the Campylobacteriales order, which contain the enteropathogenic *Campylobacter* spp. [58] and in the Catenibacterium genus which belongs to the Erysipelotrichaceae family, which we [59] and others [60] have previously shown to be enriched in the microbiota of patients with chronic HIV infection.

Gough et al performed a secondary analysis [11] of previously published data on the gut microbiome in children with SAM from Malawi [8] and Bangladesh [10]. Whereas our study compared the gut microbiota of stunted (cases) to non-stunted (controls) children, Gough et al compared severely stunted (HAZ ≤ − 3SD, cases) to stunted (HAZ > − 3SD but ≤ − 2SD, controls) children. Although, the most abundant phyla and genera in all three countries were similar, there were differences in the relative abundance of specific taxa between stunted and non-stunted children in India and between stunted and severely stunted children in Malawi and Bangladesh. For example, in the Malawi cohort, the relative abundance of *Prevotella*, *Bacteroides*, *Eubacterium and Blautia* was decreased in severely stunted, vs. stunted children whereas in the Bangladesh cohort, *Lactobacillus*, *Olsenella*, *Dorea and Bautia* were decreased in severely stunted, vs. stunted children.

More recently, Blanton et al showed that there is a causal relationship between immaturity of the gut microbiota and stunting and underweight [9] in children in the Malawi study [10] as there was with severe acute malnutrition in the Bangladesh study [50]. Secondary analysis of the gut microbiota of stunted children in Malawi and Bangladesh by Gough et al indicated that an increase in the relative abundance of Acidaminococcus was associated with lower future linear growth [11]. The study by Blanton et al found that *Ruminococcus gnavus* and *Clostridium symbiosum* from the microbiota of healthy children in Malawi promoted growth in mice transplanted with microbiota from undernourished children [9]. Our study was not designed to answer the same questions as in these studies. However, although *Ruminococcus gnavus* was present in both cases and controls, we were not able to find a differential abundance of this species between stunted and non-stunted children. Acidaminococcus was present only in the cases, but not in the controls and *Clostridium symbiosum* was not identified in either cases or controls, suggesting that there may be regional differences in the prevalence or abundance of these taxa.

The strengths of our pilot study are 1) the longitudinal design which enabled us to determine the effect of increasing age on the composition and diversity of the gut microbiota and to combine data from different ages to identify differentially abundant taxa between cases and controls 2) the conduct of the study in a well-defined community where there are no major differences in socioeconomic status, environmental factors such as water source or diet. The main weakness is the small sample size, which, although similar to the sample sizes (ranging from 6 to 10 in cases and controls) in the study by Gough et al [11], did not allow us to account for confounding factors such as antibiotic use, which occurred in both cases and controls. Nevertheless, we found significant differences in the gut microbiota of stunted compared to non-stunted children. Larger, longitudinal studies including metagenomic and metabolomic approaches to determine the compositional and functional maturation of the microbiome in children in this or other birth cohorts as well as animal studies are needed to design targeted interventions to prevent or treat this form of malnutrition.

## Supporting Information

S1 Fig Heatmap of stunting, underweight, wasting, and episodes of diarrhea and antibiotic use in cases and controls. N, no; Y, yes.
(TIF)

S2 Fig Increase in Phylogenetic Diversity (PD) index with increasing age in individual case and control children in relation to age of weaning, diarrheal episodes and antibiotic use. Color code: dark green circles = PD of controls at each time point; red circles = PD of cases at each time point. Light green squares = age at weaning; blue triangles = diarrheal episodes that were treated with antibiotics; orange triangles = diarrheal episodes that were not treated with antibiotics; grey triangles = illnesses other than diarrhea that were treated with antibiotics.
(TIF)

S3 Fig Beta diversity analysis using Principal Coordinates Analysis (PCoA) of Unifrac distances. **a:** PCoA of weighted UniFrac distances at different time points. **b:** Comparison of weighted UniFrac distances between cases and controls at different time points determined using a 2 sample t test with 1000 Monte Carlo permutations and Bonferroni corrections. * = P < 0.05.
(TIF)

S4 Fig Relative abundance of 10 most abundant genera in cases and controls at each time point. (TIF)

S1 Table Nutritional status. (DOCX)

S2 Table Relative abundance of major phyla in controls and cases at 3 monthly intervals. (DOCX)

S3 Table Alpha diversity indices in controls and cases at 3-monthly intervals. (DOCX)
