# Supplementary figures and images for: Longitudinal Analysis of the Intestinal Microbiota in Persistently Stunted Young Children in South India
Source: PLoS One. 2016 May 26;11(5):e0155405. doi: 10.1371/journal.pone.0155405 (PMC4881907; doi:10.1371/journal.pone.0155405)

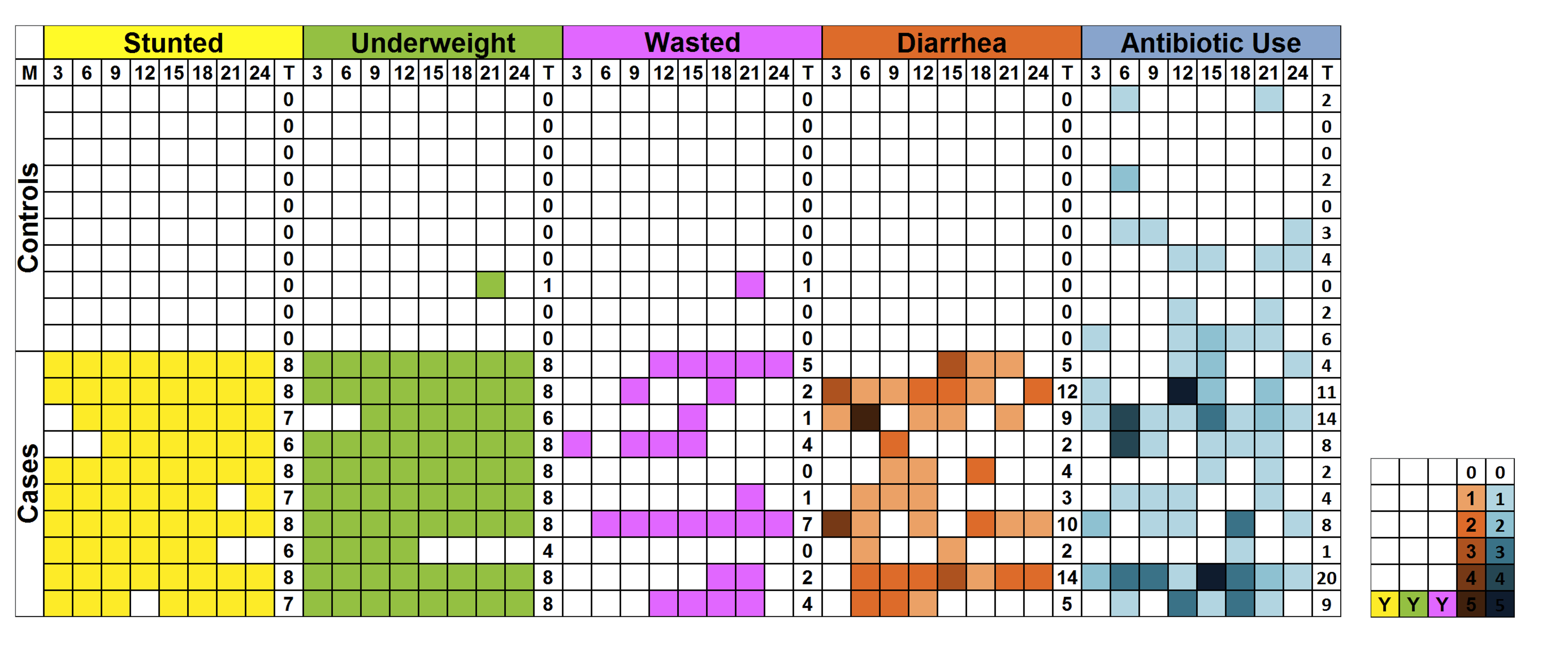

Supplement: S1 Fig — N, no; Y, yes. (TIF) [file pone.0155405.s001.tif]

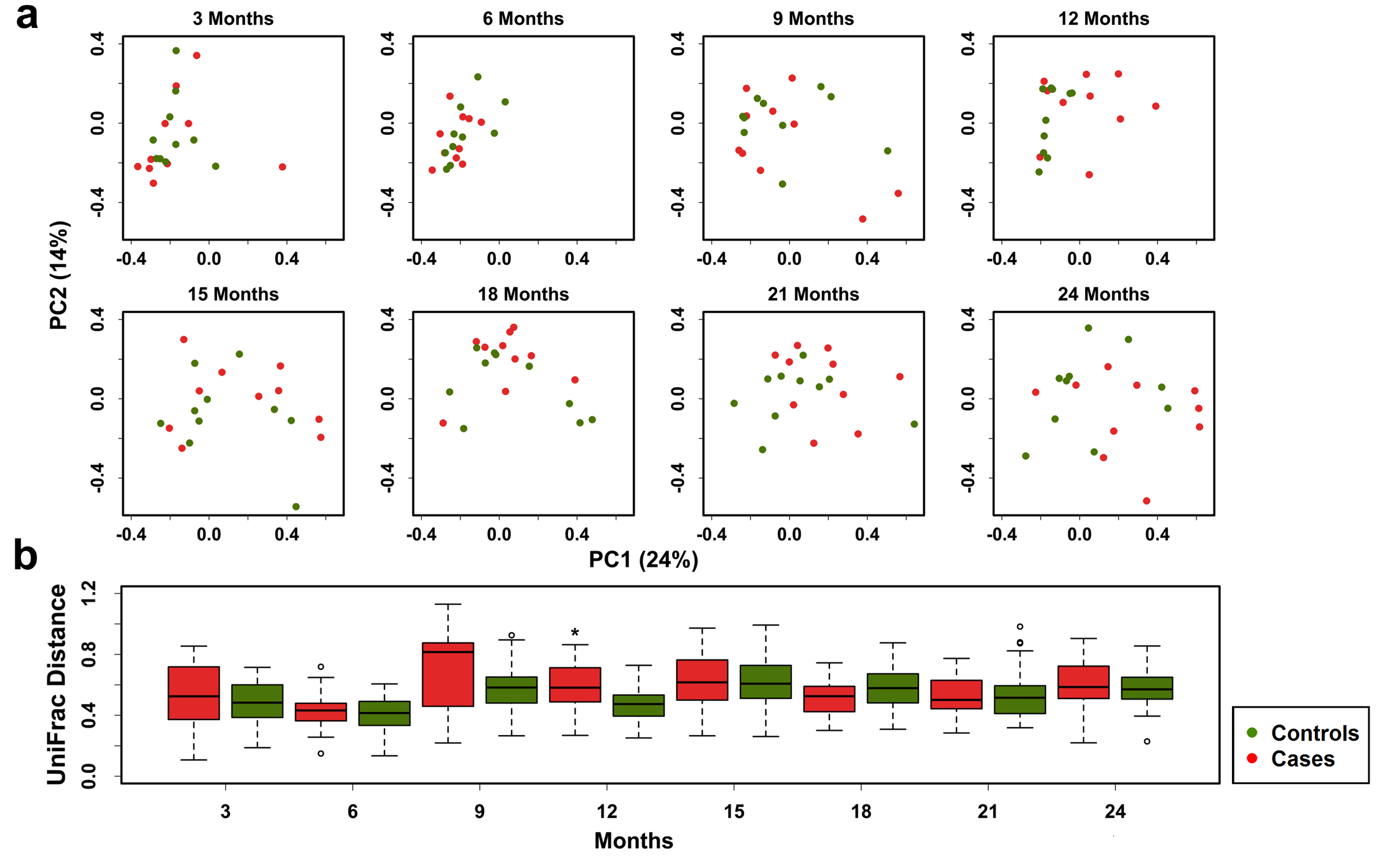

Supplement: S3 Fig — a: PCoA of weighted UniFrac distances at different time points. b: Comparison of weighted UniFrac distances between cases and controls at different time points determined using a 2 sample t test with 1000 Monte Carlo permutations and Bonferroni corrections. * = P < 0.05. (TIF) [file pone.0155405.s003.tif]

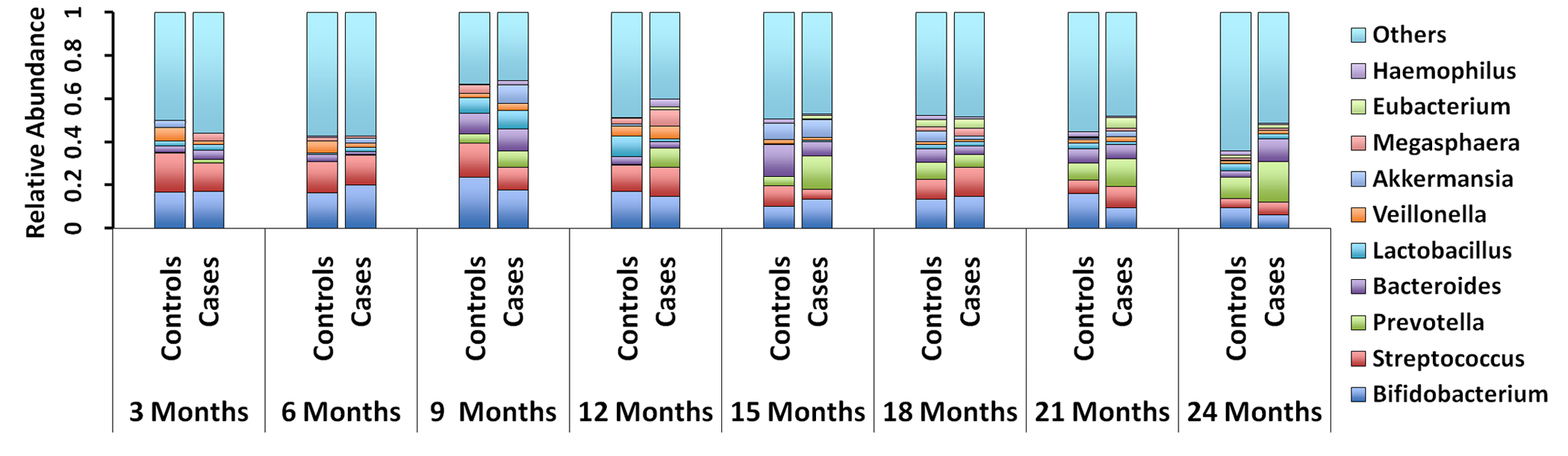

Supplement: S4 Fig — (TIF) [file pone.0155405.s004.tif]
