# Supplementary material for: Longitudinal Analysis of the Intestinal Microbiota in Persistently Stunted Young Children in South India
Source: PLoS One. 2016 May 26;11(5):e0155405. doi: 10.1371/journal.pone.0155405 (PMC4881907; doi:10.1371/journal.pone.0155405)
Supplement: S1 Table — (DOCX) [file pone.0155405.s005.docx]

**S1 Table: Nutritional status.**

**a. Anthropometry and z scores at 3-monthly intervals.**

| Age (Mths) | Weight  (Kg) | | | Height  (Cm) | | | HAZ score | | | WAZ score | | | WHZ score | | |
| --- | --- | --- | --- | --- | --- | --- | --- | --- | --- | --- | --- | --- | --- | --- | --- |
|  | **Controls** | **Cases** | **P** | **Controls** | **Cases** | **P** | **Controls** | **Cases** | **P** | **Controls** | **Cases** | **P** | **Controls** | **Cases** | **P** |
| 3 | 5.40 | 4.45 | 0.0004 | 59.7 | 55.5 | 0.0005 | -0.30 | -2.56 | <0.0001 | -0.71 | -2.88 | <0.0001 | -0.01 | -0.76 | 0.3150 |
| 6 | 7.30 | 5.20 | 0.0002 | 65.9 | 60.4 | 0.0003 | -0.29 | -2.43 | <0.0001 | -0.10 | -2.81 | <0.0001 | -0.14 | -1.50 | 0.0288 |
| 9 | 8.00 | 5.85 | 0.0002 | 71.0 | 64.4 | <0.0001 | -0.21 | -2.76 | <0.0001 | -0.44 | -2.93 | <0.0001 | -0.29 | -1.86 | 0.0283 |
| 12 | 8.70 | 6.50 | <0.0001 | 73.8 | 67.5 | 0.0002 | -0.26 | -2.89 | <0.0001 | -0.27 | -2.82 | <0.0001 | -0.04 | -1.81 | 0.0052 |
| 15 | 9.25 | 6.95 | <0.0001 | 77.2 | 71.0 | 0.0003 | -0.28 | -2.59 | <0.0001 | -0.43 | -2.86 | <0.0001 | -0.50 | -2.02 | 0.0029 |
| 18 | 10.6 | 8.55 | <0.0001 | 83.9 | 77.2 | <0.0001 | -0.80 | -2.94 | <0.0001 | -0.70 | -2.69 | <0.0001 | -0.40 | -1.67 | 0.0009 |
| 21 | 10.1 | 8.00 | 0.0003 | 82.1 | 75.5 | 0.0006 | -0.62 | -3.01 | <0.0001 | -0.92 | -2.72 | <0.0001 | -0.63 | -1.92 | 0.0015 |
| 24 | 10.6 | 8.55 | <0.0001 | 83.9 | 77.2 | <0.0001 | -0.80 | -2.94 | <**0.0001** | -0.70 | -2.69 | <0.0001 | -0.40 | -1.67 | 0.0009 |

Data are expressed as medians and analyzed by Mann-Whitney Test.

**b. Number of children with stunting, wasting and underweight at 3-monthly intervals.**

| Age  (Mths) | Stunting | | | Wasting | | | Underweight | | |
| --- | --- | --- | --- | --- | --- | --- | --- | --- | --- |
|  | **Controls** | **Cases** | **P** | **Controls** | **Cases** | **P** | **Controls** | **Cases** | **P** |
| 3 | 0 (0%) | 8 (80%) | 0.0007 | 0 (0%) | 1 (10%) | 1.0000 | 0 (0%) | 9 (90%) | 0.0001 |
| 6 | 0 (0%) | 9 (90%) | 0.0001 | 0 (0%) | 1 (10%) | 1.0000 | 0 (0%) | 9 (90%) | 0.0001 |
| 9 | 0 (0%) | 10(100%) | <0.0001 | 0 (0%) | 3 (30%) | 0.2105 | 0 (0%) | 10 (100%) | <0.0001 |
| 12 | 0 (0%) | 10 (100%) | <0.0001 | 0 (0%) | 4 (40%) | 0.0867 | 0 (0%) | 10 (100%) | <0.0001 |
| 15 | 0 (0%) | 10 (100%) | <0.0001 | 0 (0%) | 5 (50%) | 0.0325 | 0 (0%) | 9 (90%) | 0.0001 |
| 18 | 0 (0%) | 10 (100%) | <0.0001 | 0 (0%) | 5 (50%) | 0.0325 | 0 (0%) | 9 (90%) | 0.0001 |
| 21 | 0 (0%) | 8 (80%) | 0.0007 | 1 (10%) | 5 (50%) | 0.1409 | 1 (10%) | 9 (90%) | 0.0011 |
| 24 | 0 (0%) | 9 (90%) | 0.0001 | 0 (0%) | 2 (20%) | 0.4737 | 0 (0%) | 9 (90%) | 0.0001 |

Data analyzed by Fisher’s Exact Test.
