# Supplementary material for: Longitudinal Analysis of the Intestinal Microbiota in Persistently Stunted Young Children in South India
Source: PLoS One. 2016 May 26;11(5):e0155405. doi: 10.1371/journal.pone.0155405 (PMC4881907; doi:10.1371/journal.pone.0155405)
Supplement: S2 Table — (DOCX) [file pone.0155405.s006.docx]

**S2 Table: Relative abundance of major phyla in controls and cases at 3 monthly intervals.**

| Month | RA* | Controls | Cases | P |
| --- | --- | --- | --- | --- |
| 3 | Actinobacteria | 0.2045 (0.0525, 0.2803) | 0.1572 (0.0712, 0.3215) | 0.9118 |
|  | Bacteroidetes | 0.0003 (0.0001, 0.0552) | 0.0067 (0.0002, 0.0601) | 0.3248 |
|  | Firmicutes | 0.3278 (0.2075, 0.5467) | 0.3284 (0.1139, 0.4699) | 0.6842 |
|  | Proteobacteria | 0.3584 (0.1971, 0.5069) | 0.4277 (0.2050, 0.5992) | 0.5288 |
|  |  |  |  |  |
| 6 | Actinobacteria | 0.171 (0.0838, 0.2818) | 0.2125 (0.1032, 0.3313) | 0.6305 |
|  | Bacteroidetes | 0.0341 (0.0002, 0.0778) | 0.0041 (0.0009, 0.0341) | 0.3445 |
|  | Firmicutes | 0.3761 (0.2689, 0.5200) | 0.3105 (0.2241, 0.4183) | 0.3527 |
|  | Proteobacteria | 0.4028 (0.2271, 0.4814) | 0.374 (0.2423, 0.5955) | 0.9118 |
|  |  |  |  |  |
| 9 | Actinobacteria | 0.1823 (0.1177, 0.3318) | 0.1470 (0.0164, 0.3628) | 0.4813 |
|  | Bacteroidetes | 0.0947 (0.0034, 0.2405) | 0.0762 (0.0203, 0.2451) | 0.9705 |
|  | Firmicutes | 0.4749 (0.2000, 0.5511) | 0.2668 (0.0864, 0.4132) | 0.1655 |
|  | Proteobacteria | 0.1921 (0.1074, 0.2580) | 0.2038 (0.0805, 0.4895) | 0.9118 |
|  |  |  |  |  |
| 12 | Actinobacteria | 0.2245 (0.1266, 0.2727) | 0.1299 (0.0414, 0.2229) | 0.1655 |
|  | Bacteroidetes | 0.0447 (0.0081, 0.0712) | 0.1057 (0.0292, 0.2161) | **0.0433** |
|  | Firmicutes | 0.3655 (0.2310, 0.5466) | 0.5431 (0.2332, 0.5881) | 0.4359 |
|  | Proteobacteria | 0.2589 (0.1246, 0.4953) | 0.165 (0.0736, 0.3197) | 0.2799 |
|  |  |  |  |  |
| 15 | Actinobacteria | 0.08242 (0.0528, 0.2033) | 0.1795 (0.0287, 0.2316) | 0.6842 |
|  | Bacteroidetes | 0.1059 (0.0741, 0.3035) | 0.1966 (0.0615, 0.3858) | 0.7959 |
|  | Firmicutes | 0.3006 (0.1335, 0.4956) | 0.3037 (0.2363, 0.4182) | 0.9118 |
|  | Proteobacteria | 0.255 (0.1136, 0.4332) | 0.1507 (0.0668, 0.3136) | 0.3930 |
|  |  |  |  |  |
| 18 | Actinobacteria | 0.1621 (0.0798, 0.2893) | 0.2066 (0.1151, 0.2643) | 0.9705 |
|  | Bacteroidetes | 0.0743 (0.0185, 0.3634) | 0.09787 (0.0130, 0.1631) | 0.9118 |
|  | Firmicutes | 0.391 (0.2577, 0.5388) | 0.5058 (0.4057, 0.6407) | 0.1431 |
|  | Proteobacteria | 0.1465 (0.1035, 0.2162) | 0.09232 (0.0196, 0.1668) | 0.123 |
|  |  |  |  |  |
| 21 | Actinobacteria | 0.1763 (-.0516, 0.3137) | 0.1351 (0.0738, 0.2136) | 0.5288 |
|  | Bacteroidetes | 0.1032 (0.0648, 0.1745) | 0.1932 (0.0867, 0.3322) | 0.2176 |
|  | Firmicutes | 0.3436 (0.2707, 0.5488) | 0.459 (0.3912, 0.5580) | 0.4813 |
|  | Proteobacteria | 0.2221 (0.1260, 0.2964) | 0.1203 (0.0578, 0.2802) | 0.2799 |
|  |  |  |  |  |
| 24 | Actinobacteria | 0.1093 (0.0687, 0.1811) | 0.0453 (0.0236, 0.1263) | 0.063 |
|  | Bacteroidetes | 0.0532 (0.0315, 0.2666) | 0.2831 (0.1514, 0.5604) | *0.0524* |
|  | Firmicutes | 0.4197 (0.2925, 0.5863) | 0.4012 (0.2249, 0.4916) | 0.7959 |
|  | Proteobacteria | 0.1914 (0.0888, 0.4418) | 0.1886 (0.0709, 0.3201) | 0.7394 |
| *RA, relative abundance, Median (IQR). Statistically significant (<0.05) P values are in bold and P values trending towards statistical significance are in italics | | | | |
