# Supplementary material for: Longitudinal Analysis of the Intestinal Microbiota in Persistently Stunted Young Children in South India
Source: PLoS One. 2016 May 26;11(5):e0155405. doi: 10.1371/journal.pone.0155405 (PMC4881907; doi:10.1371/journal.pone.0155405)
Supplement: S3 Table — (DOCX) [file pone.0155405.s007.docx]

**S3 Table: Alpha diversity indices in controls and cases at 3-monthly intervals.**

| Month | Diversity Index* | Controls | Cases | P |
| --- | --- | --- | --- | --- |
| 3 | Chao | 5.61 (5.41, 5.77) | 5.58 (5.34, 5.71) | 0.7394 |
|  | Equitability | -0.84 (-0.86, -0.82) | -0.77 (-0.93, -0.67) | 0.1655 |
|  | Observed OTUs | 5.09 (4.94, 5.31) | 5.14 (4.79, 5.27) | 0.9705 |
|  | PD | 2.47 (2.38, 2.68) | 2.60 (2.27, 2.71) | 0.9705 |
|  | Shannon | 1.15 (1.08, 1.21) | 1.24 (0.99, 1.37) | 0.315 |
|  |  |  |  |  |
| 6 | Chao | 5.80 (5.52, 5.88) | 5.72 (5.42, 5.91) | 0.6305 |
|  | Equitability | -0.78 (-0.85, -0.70) | -0.79 (-1.04, -0.74) | 0.393 |
|  | Observed OTUs | 5.36 (5.09, 5.45) | 5.20 (5.00, 5.43) | 0.5966 |
|  | PD | 2.77 (2.50, 2.85) | 2.67 (2.37, 2.91) | 0.5787 |
|  | Shannon | 1.26 (1.17, 1.37) | 1.23 (0.93, 1.30) | 0.3527 |
|  |  |  |  |  |
| 9 | Chao | 5.81 (5.66, 5.99) | 5.86 (5.65, 6.00) | 1 |
|  | Equitability | -0.67 (-0.94, -0.58) | -0.72 (-1.05, -0.65) | 0.2799 |
|  | Observed OTUs | 5.42 (5.21, 5.60) | 5.45 (5.22, 5.58) | 0.9118 |
|  | PD | 2.77 (2.61, 2.87) | 2.81 (2.60, 2.99) | 0.7394 |
|  | Shannon | 1.41 (1.05, 1.49) | 1.34 (1.00, 1.43) | 0.393 |
|  |  |  |  |  |
| 12 | Chao | 6.00 (5.81, 6.35) | 6.08 (5.96, 6.25) | 0.4359 |
|  | Equitability | -0.75 (-0.90, -0.63) | -0.71 (-0.83, -0.59) | 0.6305 |
|  | Observed OTUs | 5.55 (5.35, 5.90) | 5.63 (5.53, 5.79) | 0.4961 |
|  | PD | 3.01 (2.83, 3.25) | 3.11 (2.82, 3.19) | 0.6305 |
|  | Shannon | 1.35 (1.15, 1.47) | 1.38 (1.27, 1.50) | 0.5288 |
|  |  |  |  |  |
| 15 | Chao | 6.27 (5.83, 6.47) | 6.31 (5.95, 6.59) | 0.5787 |
|  | Equitability | -0.65 (-0.82, -0.54) | -0.67, -0.81, -0.55) | 0.8534 |
|  | Observed OTUs | 5.83 (5.45, 6.02) | 5.87 (5.50, 6.15) | 0.7394 |
|  | PD | 3.16 (2.87, 3.29) | 3.16 (2.95, 3.47) | 0.7959 |
|  | Shannon | 1.48 (1.22, 1.63) | 1.51 (1.24, 1.59) | 0.9118 |
|  |  |  |  |  |
| 18 | Chao | 6.39 (6.31, 6.59) | 6.37 (6.06, 6.71) | 0.9118 |
|  | Equitability | -0.63 (-0.68, -0.55) | -0.59 (-0.71, -0.50) | 0.9118 |
|  | Observed OTUs | 5.97 (5.84, 6.12) | 5.90 (5.62, 6.25) | 0.7959 |
|  | PD | 3.29 (3.22, 3.54) | 3.36 (3.02, 3.57) | 0.8534 |
|  | Shannon | 1.51 (1.45, 1.65) | 1.53 (1.39, 1.71) | 0.9118 |
|  |  |  |  |  |
| 21 | Chao | 6.33 (5.85, 6.76) | 6.61 (6.26, 6.75) | 0.393 |
|  | Equitability | -0.85 (-0.63, -0.45) | -0.58 (-0.63, -0.46) | 0.9705 |
|  | Observed OTUs | 5.95 (5.46, 6.36) | 6.16 (5.82, 6.31) | 0.6305 |
|  | PD | 3.27 (2.81, 3.61) | 3.53 (3.21, 3.60) | 0.393 |
|  | Shannon | 1.53 (1.47, 1.77) | 1.60 (1.49, 1.75) | 0.7394 |
|  |  |  |  |  |
| 24 | Chao | 6.48 (6.28, 6.63) | 6.31 (6.02, 6.63) | 0.4813 |
|  | Equitability | -0.60 (-0.66, -0.51) | -0.54 (-073, -0.52) | 0.8534 |
|  | Observed OTUs | 6.03 (5.79, 6.23) | 5.92 (5.55, 6.19) | 0.5288 |
|  | PD | 3.37 (3.16, 3.50) | 3.30 (3.0, 3.48) | 0.5787 |
|  | Shannon | 1.56 (1.50, 1.66) | 1.61 (1.39, 1.68) | 1 |
